# Inhibition of Chitosan Ice Coating on the Quality Deterioration of Quick-Frozen Fish Balls during Repeated Freeze–Thaw Cycles

**DOI:** 10.3390/foods12040717

**Published:** 2023-02-07

**Authors:** Lixin Chang, Ying Li, Xue Bai, Xiufang Xia, Weidong Xu

**Affiliations:** 1 College of Food Science, Northeast Agricultural University, Harbin 150030, China; 2 Office of Student Work, Heilongjiang Agricultural Engineering Vocational College, Harbin 150088, China

**Keywords:** chitosan, ice coating, fish balls, freeze–thaw cycles, quality deterioration

## Abstract

Chitosan ice coating’s properties and its inhibitory effect on the quality deterioration of quick-frozen fish balls during repeated freeze–thaw cycles were investigated. When the chitosan (CH) coating concentration increased, the viscosity and ice coating rate increased, while water vapor permeability (WVP), water solubility, and transmittance decreased, and 1.5% CH was regarded as the excellent coating to apply to freeze–thaw quick-frozen fish balls. As the freeze–thaw cycles increased, the frost production, total volatile base nitrogen (TVB-N) values, and free water content of all of the samples increased significantly (*p* < 0.05), and the whiteness values, textural properties, and water-holding capacity (WHC) decreased. Freeze–thaw cycles expanded the aperture between the muscle fibers and the occurrence of crystallization and recrystallization between cells increased, damaging the original intact tissue structure, which were confirmed by SEM and optical microscopy. Compared with the untreated ones, the frost production, free water, and TVB-N of the samples with 1.5% CH decreased during 1, 3, 5, and 7 cycles, and were reduced by 23.80%, 32.21%, 30.33%, and 52.10% by the 7th cycle. The WHC and texture properties showed an increasing trend during the freeze–thaw cycles. Therefore, the chitosan ice coating effectively inhibited the quality deterioration by reducing water loss, the occurrence of ice crystallization and recrystallization, and the pores of the samples.

## 1. Introduction

Fish balls are made from freshwater fish from coastal areas by squeezing and tumbling, and are well-liked by customers because of their distinct texture and rich nutritional value [1]. However, because of their high protein and moisture content, fish products are vulnerable to spoiling caused by the development and growth of microorganisms, as well as the oxidation of proteins and fats, which cannot meet the consumption requirements of mainlanders. Thus, quick freezing is used to preserve fish products [2]. Throughout the quick freezing process, the generation of small ice crystals can greatly reduce muscle damage [3], while microbial replication and fat oxidation are inhibited, thus effectively delaying the spoilage and maintaining the quality of fish products [4,5].

However, the imperfection of cold chain technology leads to the temperature fluctuations of quick-frozen fish balls still occurring during circulation [6], storage, and sales [2], making fish balls in a continuous cycle of freeze–thaw. Repeated freeze–thaw cycles cause ice crystals to recrystallize, destroy the muscle tissue, and increase water loss, which speeds up the growth and reproduction of microorganisms and causes the quality of fish balls to decline. To postpone the deterioration caused by freeze–thaw cycles on quick-frozen fish balls, new freezing technologies combine physical field-assisted technology such as ultrasonic technology [7], electromagnetic [8], high pressure [9], and radiofrequency [10]. However, such freezing technology requires higher instrumentation and cost, which is not conducive to the industrial mass production of quick-frozen fish balls. Compared with this technology, the ice coating is widely recognized for its ease of operation, lower equipment costs, and its ability to produce favorable preservation effects. Chitosan (CH), sourced from shrimp and crab shells, is suitable for the application of ice coating technology on the surface of quick-frozen fish balls because of its unique assets such as its biodegradability, non-toxicity, biocompatibility, and anti-fungal properties [11,12]. At present, chitosan has been widely used in food packing and preservation; however, CH as an ice coating to inhibit the quality deterioration of food during cold chain transportation has not yet received much attention.

Therefore, this study aimed to develop new ice coating materials to investigate its inhibitory effect on the quality deterioration of quick-frozen fish balls during repeated freeze–thaw cycles by studying (1) chitosan ice coating’s properties; (2) the influence of chitosan ice coating on the frost production, water holding capacity, textural properties, whiteness, and moisture distribution; (3) the changes in the microstructure and TVB-N values on the surface of quick-frozen fish balls, which has certain theoretical significance to enhance the storability and shelf life of fish balls.

## 2. Materials and Methods

### 2.1. Materials

Glutaraldehyde and paraformaldehyde were purchased from Sigma Chemical Co. (St. Louis, MO, USA). Chitosan was purchased from Hebei Lihua Biotechnology Co. (Shijiazhuang, China). All other chemicals (glacial acetic acid et al.) were obtained from Wantai Biomedicals Inc. (Harbin, China) and were at least of analytical grade.

### 2.2. Sample Preparation

#### 2.2.1. Chitosan Ice Coating

A dispersion of CH at 0.5, 1.0, 1.5, and 2.0 % was dissolved in glacial acetic acid to 1 % and stirred magnetically until a homogeneous and transparent liquid was obtained, then degassed in ultrasonication (224 W, 15 min) and set aside; the cleaned and dry-cooled slides were completely immersed in different concentrations of CH solutions, slowly taken out after 5 min, dried in an oven (Foshan Winsea Precision Instruments Co., Guangdong, China) at 40 °C for 1 h, and cooled at room temperature [13]. After cooling, the coatings were removed and stored in a 4 °C refrigerator for further operations.

#### 2.2.2. Fish Balls

Fresh mirror carp (2–3 kg) was obtained from Haoyouduo supermarket (Harbin, Heilongjiang, China) and was promptly killed by the skilled clerk with a blunt object. After being de-headed, de-tailed, and de-gutted, they were delivered to the laboratory under frozen conditions within 1 h. Upon arrival, the skin and the black membrane on the abdomen of the fish were removed, and the flesh was picked off and placed in the 4 °C refrigerators for subsequent operations. The removed flesh was rinsed and chopped into small cubes (2 × 2 × 2 cm^3^), then added to the meat grinder to form minced fish, and the weighed. The obtained surimi paste after 8 min of grinding was ground for 8 min with 2% (*w*/*w*) edible salt, finally combined thoroughly for 5 min with the additional food ingredients and additives (8% ice water, 10% corn starch) [14]. All processes were performed at a temperature below 4 °C. The fish balls made from surimi paste were submitted to flesh freezing for 24 h at −30 °C in a refrigerator and were wrapped in plastic wrap to obtain quick-frozen fish balls. Quick-frozen fish balls were immersed in a 1.5% CH solution (10 s), removed, and drained. The samples without coating served as the control group before the first freeze–thaw cycle. The packed fish balls were frozen at −18 °C for 7 d, then thawed at 4 °C for 4 h to increase the sample’s internal temperature to 0–4 °C. This process was counted as one freeze–thaw. The samples from each treatment were exposed to experimental analysis, which was conducted for the 0, 3, 5, and 7 freeze–thaw treatments.

### 2.3. Properties of Chitosan Ice Coating

#### 2.3.1. Viscosity

Different concentrations of CH solutions were measured at room temperature according to Cho et al. [15], with slight modifications, using a viscometer (LVDV-E Viscometer, Brookfield Engineering USA Ltd., Massachusetts., MA, USA).

#### 2.3.2. Ice Coating Rate

The ice coating rate was measured by Sundararajan et al. [16]. The quick-frozen fish balls were weighed and labeled as M_1_ (g) in an operating environment at 4 °C, and immersed in CH solutions of different concentrations. After soaking for 10 s, they were taken out, drained, and weighed as M_2_. The calculation formula is as follows:

(1) $$\mathrm{Ice}\;\mathrm{coating}\;\mathrm{rate}\;(\%)=\frac{M_{2}-M_{1}}{M_{2}}\;\times \;100$$

#### 2.3.3. Water Vapor Permeability (WVP)

The WVP value was measured according to Tavares et al. [12], with slight modifications. The experiment was carried out in an incubator at 25 °C and 70% RH. The specific method was as follows: Weigh 3 g of anhydrous CaCl_2_ (cover 0.46 cm from the bottom) that has been pulverized and dried in advance, and spread it evenly at the bottom of a 10 mL centrifuge tube (without lid, 1 cm diameter). Cut the CH coatings with different concentrations into squares of 2 × 2 cm^2^. After measuring their weight and thickness, cover the coating to the mouth of the centrifuge tube with the help of a sealing film. Place the centrifuge tube on an analytical balance (Shanghai Mettler-Toledo Instruments Co., Shanghai, China) and weigh it. Then weigh it every 12 h until the overall weight of the centrifuge tube is basically stable. The calculation formula is as follows:

(2) $$\mathrm{WVP}\;(g\cdot m^{-1}S^{-1}{\mathrm{Pa}}^{-1})=\frac{\Delta m\times d}{A\times \Delta t\times \Delta p}$$

∆m: Weight difference of anhydrous CaCl_2_ (g); d: thickness of coating (m); A: coating area (m^2^); ∆t: time of measurement (s); ∆p: partial water vapor pressure difference on both sides of the coating (Pa).

#### 2.3.4. Water Solubility

Water solubility was extracted according to the method of Hosseini et al. [13]. The CH coatings of different concentrations were cut into 2 × 2 cm^2^ squares, dried for 2 h in a 105 °C oven before being weighed, and marked as M_3_ (g); the dried coatings were placed at the bottom of 30 mL of distilled water so that the coatings were completely immersed in water and left under continuous shaking conditions for 24 h. After filtration, the undissolved coating was obtained, which was dried (105 °C, 2 h), weighed, and labeled as *M_4_*. The calculation formula is as follows:

(3) $$\mathrm{Water}\;\mathrm{solubility}\;\%=\frac{M_{3}-M_{4}}{M_{3}}\;\times \;100$$

#### 2.3.5. Transmittance

The coating was cut into rectangles (12 × 40 mm) and tightly attached to the inside of the cuvette, and the absorbance values were measured with a UT-1800 UV/VIS spectrophotometer (AuCy Scientific Instrument Co., Ltd., Shanghai, China) at a 500 nm wavelength using an empty cuvette as a control group and labeled as A [17]. The calculation formula is as follows:

(4) $$\mathrm{Transmittance}\;(\%)=\frac{1}{10^{A}}\;\times \;100$$

### 2.4. Influence of Chitosan Ice Coating on the Quality of Fish Balls

#### 2.4.1. Frost Production

Frost production was measured to follow the method of Urquiola et al. [18], with some modifications. Under the operating environment of 4 °C, the quick-frozen fish balls with or without 1.5% CH ice coating were accurately weighed (with an accuracy of 0.0001) and marked as M_5_ (g). Weighed fish balls were kept in a refrigerator at a temperature of –18 °C. After different freeze–thaw cycles, the frost on the surface of the quick-frozen fish balls was quickly scraped off with a pre-cooled sharp double-sided blade and weighed to mark it as M_6_ (g). The calculation formula is as follows:

(5) $$\mathrm{Frost}\;\mathrm{production}\;(\%)=\frac{M_{6}-M_{5}}{M_{5}}\;\times \;100$$

#### 2.4.2. Water-Holding Capacity (WHC)

Thawing loss was determined based on the approach of Bai et al. [19]. The sample’s weight was measured before thawing (M_7_) and after thawing (M_8_). The calculation formula is as follows:

(6) $$\mathrm{Thawing}\;\mathrm{loss}\;(\%)=\frac{M_{7}-M_{8}}{M_{7}}\;\times \;100$$

The thawed fish balls were weighed and marked as M_9_, placed in a cooking bag, and heated until the fish balls’ internal temperature reached 75 °C in an 85 °C water bath. The water on the surface of the fish balls was then quickly blotted dry using filter paper, and the fish balls were weighed and designated as M_10_ (g) [20]. The calculation formula is as follows:

(7) $$\mathrm{Cooking}\;\mathrm{loss}\;(\%)=\frac{M_{9}-M_{10}}{M_{9}}\;\times \;100$$

Cut 2 g of quick-frozen fish balls, wrap them in filter paper, put them in a 10 mL centrifugation tube, and centrifuge them for 10 min. Weigh the fish balls before and after centrifugation and mark them as M_11_ (g) and M_12_ (g) [21]. The calculation formula is as follows:

(8) $$\mathrm{Centrifugal}\;\mathrm{loss}\;(\%)=\frac{M_{11}-M_{12}}{M_{11}}\;\times \;100$$

#### 2.4.3. Texture Properties

Texture properties were determined by following the approach of Gao et al. [22]. After the determination of cooking loss, samples were cut into cylinders from the center after being cooled to room temperature, and the elasticity, cohesion, and chewiness of the fish balls were measured twice in compression using a texture analyzer (Stable Micro System, TA: XT2i, London, UK) P/50 probe (50 mm diameter). The capacity of the load cell of the equipment was 5 kg. The measurement parameters were as follows: pre-measurement velocity: 5 mm/s; mid-measurement velocity: 1 mm/s; post-measurement velocity: 5 mm/s; strain: 50%; time: 5 s; trigger force: 5 g.

#### 2.4.4. Whiteness

The whiteness value was determined according to the method of Jia et al. [23]. The color of the fish balls was measured by a ZE-6000 color difference meter (Denshoku Corporation, Tokyo, Japan). The brightness value (L*), redness value (a*), and yellowness value (b*) of the fish balls were measured and recorded. The calculation formula for the whiteness value is as follows:

(9) $$\mathrm{Whiteness}=100\;-\;\sqrt{{\left(100\;-L*\right)}^{2}{+a*}^{2}{+b*}^{2}}$$

#### 2.4.5. Low Field Nuclear Magnetic Resonance (LF-NMR) and Nuclear Magnetic Resonance Imaging

A minor adjustment was made according to the technique of Du et al. [2]. The thawed (4 °C) fish balls were cut into cylinders and placed in a cylindrical NMR tube for measurement, with the sample held tightly against the NMR tube wall. The transverse relaxation time (*T*_2_) was measured following the normalization of the raw data.

Proton density profiles of the mirror carp meat were obtained by spin-echo (SE) imaging sequences using a MesoMR23-060V-1 NMR analyzer (Newman Analytics Ltd., Suzhou, China) according to the method of Nian et al. [24], with slight modifications. The fish balls were wrapped with cling film and placed in an NMR tube, and subsequently placed in an MR-60 MRI machine to be imaged and analyzed. The proton density maps were obtained through preliminary processing with Newman Ver 1.0 software (Beijing Sprite Technology Development Co., Beijing, China).

#### 2.4.6. Microstructure

Scanning electron microscope (Hitachi Japan, Inc., Hitachi, Japan) was measured following the method of Lan et al. [25], with some modifications. The geometric centers of the micro-frozen fish balls were cut into small cubes (0.5 × 0.5 × 0.5 cm^3^) using a sharp double-sided blade to ensure as much as possible that one cut was formed. The samples were dehydrated and put in a 20 °C refrigerator for 30 min, and finally dried in a freeze-dryer for 4 h, and observed at an accelerating voltage of 5 KV and magnification of 500× under SEM.

#### 2.4.7. Ice Crystal Morphology

The observation of the ice crystal morphology of quick-frozen fish balls was performed based on the method of Sun et al. [26]. Quick-frozen fish balls were quickly sliced into thin slices (0.5 × 0.5 × 1 cm^3^) by a pre-cooled sharp double-sided blade, then fixed in 4% paraformaldehyde for 24 h and dehydrated in anhydrous ethanol for 2 h under room temperature following fixation. The obtained samples were immersed in a mixture of 50% toluene and 50% anhydrous ethanol overnight (12 h) and eluted in toluene for 4 h after removal. The dewaxed, eluted, and water-washed samples were stained with eosin and observed under an optical microscope equipped with a digital camera (Nikon DS-5M, Nikon, Japan). Ice crystal diameters were analyzed by Image-Pro Plus software (Media Contrbernetics, Silver Spring, MD, USA).

#### 2.4.8. Total Volatile Base Nitrogen (TVB-N)

The TVB-N values were calculated using the method of Sun et al. [27], with slight modifications. First, 10 g of crushed quick-frozen fish balls were added to 100 mL of distilled water, shaken at 4 °C for 30 min, and filtered. After filtration, 5 mL of the filtrate was mixed with an equal volume of 10 g/L oxidase (MgO) and distilled for 5 min using a Kjeldahl nitrogen tester. The absorbent solution was titrated with 0.01 mol/L of standard hydrochloric acid titrant solution and the endpoint was blue-purple. The calculation for the formula is as follows:

(10) $$\mathrm{TVB}-N\;(\mathrm{mg}/100\;g)=\frac{{(V}_{1}-V_{2})\;\times \;c\;\times \;14}{m\;\times \;5/100}\;\times \;100$$

V_1_: hydrochloric acid standard solution’s volume consumed by the sample solution for determination (mL); V_2_ hydrochloric acid standard solutions consumed by the blank reagent (mL); c: hydrochloric acid standard solution’s molar concentration (mol/L); m of the sample’s mass (g).

### 2.5. Statistical Analysis

Data were expressed as mean ± standard deviation and analyzed with SPSS 25.0 software (SPSS Inc., Chicago, IL, USA). The significance of the main effects was determined by one-way analysis of variance (ANOVA) and Tukey’s multiple comparisons with a 95% confidence interval (*p* < 0.05). Each set of samples was set up in three parallel groups and the average value was the result obtained.

## 3. Results and Discussion

### 3.1. Properties of Chitosan Ice Coating

#### 3.1.1. Viscosity

Table 1 the viscosity of the coating increased with increasing the CH concentration, which was attributed to the mutual aggregation and cross-linking of molecules in the solution, reducing the fluidity and improving the adhesion of the solution. This result was similar to the finding that the rising concentration of chitosan (1–2.5%) led to an increase in the viscosity of the solution [28]. A CH solution of a high viscosity (>1000 cP) would form over the thick ice coating layer and produce a high ice coating rate of 31.9% (>14%) at 2.0% CH on the surface of the fish balls, seriously affecting the color appearance and edible quality of the product. At 0.5% and 1.0% CH, the solutions with low viscosity (<100 cP) produced ice coating rates of 2.9% and 5.2%, respectively, which did not meet the relevant regulations for the ice coating rate (3.1.2) and thus failed to achieve an excellent preservation effect [29]. Therefore, 1.5% CH solution was optimal to be used as the ice coating.

#### 3.1.2. Ice Coating Rate

The ice coating rates of CH with different concentrations on the quick-frozen fish balls are shown in Table 1. With the increase in CH concentration, the ice coating rate showed an increasing trend, up to 31.9% at 2.0% CH. This phenomenon indicated that the ice coating layer had grown thicker, which was attributed to the increasing viscosity. The EAEU Technical Regulation (TR) 040/2016 restricted the amount of glaze (ice) in the fish products: the total weight for unbroken fish products should not exceed 14% [30,31,32]. Vanhaecke et al. [33] reported 6% to 10% glazing was found to be effective for protecting the fillets from oxidation and quality deterioration. When the CH concentration was 0.5%, 1.0%, and 1.5%, the ice coating rate was in accordance with the relevant regulations.

#### 3.1.3. Water Resistance

The water resistance of the coating could be characterized by the WVP value and water solubility. With the increase in CH concentration (c), the WVP of the coating showed a trend of firstly decreasing and then increasing, and obtained the lowest value at 1.5% CH (Table 1). The decreasing WVP might be explained because the coating structure formed by hydrogen bonds between CH molecules became more compact with the increasing concentration (c < 1.5%). At high concentrations (c > 1.5%), irregular agglomeration was generated between too many CH molecular chains, and a large number of hydrophilic groups in CH were exposed, raising the coating’s WVP value. Ren et al. [34] also conducted a similar study, where the WVP value of film containing chitosan decreased as the concentration of chitosan increased.

The water solubility of the coating decreased significantly (*p* < 0.05) with increasing the CH concentration (Table 1). This phenomenon might be explained by the fact that the increasing solution concentration resulted in more insoluble substances in the coating and higher compactness of the coating structure. Applying an ice coating with a low water solubility is more conducive to blocking the migration and loss of water inside the frozen fish ball. This was in accordance with the study by Hosseini et al. [13] that water solubility of fish gelatin-based films decreased as a result of increasing the chitosan concentration.

#### 3.1.4. Transmittance

Transmittance is an important indicator to characterize the coating transparency [17]. As shown in Table 1, the transmittance of the CH coating decreased with the increase in solution concentration, which was caused by the increasing solid content. The color and appearance of fish balls were improved by the excellent transmittance. Roy et al. [35] found that chitosan coating with the addition of quercetin-loaded chitosan nanoparticles exhibited pale yellow with > 83% transmittance and superior transparency. In conclusion, the coating formed after ice coating with concentrations of 0.5%, 1.0%, and 1.5% CH ice coating solution had a high transmittance and would not affect the color and appearance of the product.

### 3.2. Influence of Chitosan Ice Coating on the Quality of Fish Balls by Freeze–Thaw Cycles

#### 3.2.1. Frost Production

**Figure 1:**
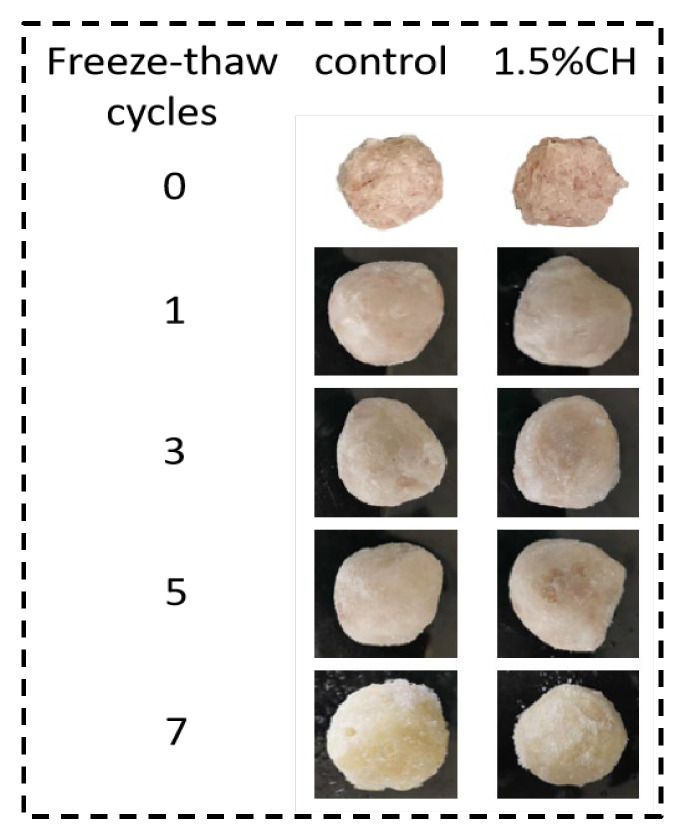
Sample diagram of frost production on the surface of the quick-frozen fish balls.

Temperature fluctuations during storage cause frost production on the surface of quick-frozen fish balls, affecting the texture and color of the fish balls. The effect of 1.5% CH coating on the amount of surface frost production and the sample appearances on quick-frozen fish balls are shown in Table 2 and Figure 1. The amount of surface frost production increased significantly (*p* < 0.05) with the increasing number of freeze–thaws for both groups of samples. This was mainly due to the continuous alternation of ice crystal formation and water loss during the freeze–thaw cycle, with the lost water forming ice on the surface and frost in contact with water vapor.

In contrast with the control group, the surface frost production of the samples coated with 1.5% CH was reduced by 91.18%, 54.55%, 48.52%, and 40.16% during 1, 3, 5, and 7 cycles, respectively. Frost production of the ice-coated sample only decreased by 54.55% in the third cycle, which indicated that significant degradation in the quality of quick-frozen fish balls was observed in the first three freeze–thaw cycles. Similarly, Meléndez-Pérez et al. [36] showed that film-wrapped samples showed a 60% reduction in frost amount at 7 days of frozen storage compared with the control group. In the meanwhile, with increasing the freeze–thaw cycles, the inhibition effect of 1.5% CH on the quality deterioration of quick-frozen fish balls was weakened. On the one hand, this phenomenon may be attributed to the free hydroxyl group in CH molecules combining with water molecule, converting the free water into immobile water and keeping the water inside the quick-frozen fish ball again to a greater extent; on the other hand, the coating adhered to the surface of the fish ball to form a barrier layer, preventing water loss [37].

#### 3.2.2. Water-Holding Capacity (WHC)

##### Thawing Loss

Thawing loss is a significant indicator for determining the degree of WHC and nutrient loss of quick-frozen fish balls [38]. The effect of 1.5% CH coating on the thawing loss of quick-frozen fish balls during freeze–thaw cycles is shown in Table 2. Thawing loss increased with the increase in freeze–thaw cycles for both groups of samples. This was mainly due to the migration of intracellular water outside the cells during storage, with crystallization and recrystallization occurring in the muscle tissue. The growing ice crystals continuously squeezed the muscular tissue of the fish balls, causing a large amount of juice loss during thawing [39]. This was exemplified in the work undertaken by Pan et al. [40], who showed that the mechanical damage caused by recrystallization during repeated freeze–thaw cycles led to a significant increase in thawing losses in quick-frozen pork patties.

Compared with the control group at the same freeze–thaw cycle, in the 1st, 3rd, 5th, and 7th freeze–thaw cycles, thawing loss was reduced by 16.09%, 16.01%, 24.77%, and 29.50% in the CH-coated samples, respectively. This phenomenon was because the CH coating can be used as a protective barrier to protect against the dehydration of quick-frozen fish balls [41]. A similar study had shown that chitosan with greater bio adhesive properties made it easier to adhere to tissues in order to improve meat products’ WHC [42].

##### Cooking Loss

The cooking loss exhibited a similar tendency to the thawing loss during freeze–thaw cycles (Table 2). This phenomenon was because the network structure looseness and protein damage reduced the ability to bind the proteins to water, resulting in an increase in cooking loss [43].

Before the first cycle, there was no remarkable difference between the ice-coated treated samples and the control group. However, the cooking loss of 1.5% CH-coated samples was significantly lower than that of the control group at the 1st, 3rd, 5th, and 7th cycles. This resulted from the development of a barrier layer on the surface of quick-frozen fish balls by CH coating, with an excellent water retention effect, which effectively inhibited the loss of water and nutrients inside the samples during the cooking process. A similar study reported that the improved WHC of rosemary extract combined with chitosan-coated mori signified the ability of chitosan to effectively bind water [44].

##### Centrifugal Loss

Centrifugal loss is the ability of quick-frozen fish balls to retain water in the microstructure when subjected to external forces, and is one of the most important indicators to measure the closeness of the network structure of the sample. As shown in Table 2, the centrifugal losses of both groups of samples firstly increased significantly (*p* < 0.05) and then remained stable when increasing the freeze–thaw cycles. This was due to the high internal moisture content of the sample at the beginning of the freeze–thaw cycle, which led to a sharp increase in centrifugal losses, followed by a slow increase at the end of the freeze–thaw cycle, owing to a decrease in the free water content.

At the same freeze–thaw cycle, the centrifugal loss of the 1.5% CH-coated samples was significantly lower than that of the control group. During 1, 3, 5, and 7 cycles, the centrifugal loss after CH coating was reduced by 17.66%, 24.45%, 21.40%, and 22.95% compared with the control group, respectively. This phenomenon suggests that the quick-frozen fish balls treated with 1.5% CH showed excellent water retention under different freeze–thaw cycles due to the strong water resistance of the CH ice coating, and the most significant inhibition occurred in the third freeze–thaw cycle.

#### 3.2.3. Texture Properties 

Textural properties are important parameters for evaluating the quality characteristics of surimi products, which can indirectly reflect the structural integrity of fish meat myofibrillar protein (MP) and the tightness of its binding with water molecules and other components [45]. Table 3 shows the changes in springiness, cohesiveness, and chewiness in quick-frozen fish balls for two groups of samples with the increase in freeze–thaw cycles. With the freeze–thaw cycles increased, the springiness, cohesiveness, and chewiness of all of the samples decreased. This result was caused by the freezing of water molecules inside the fish ball to generate ice crystals with the increase in freeze–thaw cycles, which caused the extrusion of muscle cells and irreversible mechanical damage, damaging the gel properties of the fish ball [46]. Lv and Xie [47] also reported that freeze–thaw cycles led to a reduction in the cuttlefish flesh’s hardness, flexibility, and masticatory qualities.

The springiness, cohesiveness, and chewiness of the samples with the 1.5% CH ice coating increased by 41.27%, 78.72%, 95.12%, and 84.62%; 90%, 107.70%, 104.17%, and 77.27%; and 11.28%, 16.94%, 18.10%, and 26.26% during cycles 1, 3, 5, and 7. This phenomenon showed that the excellent water resistance of CH inhibited the reduction in WHC and effectively prevented the disintegration of the internal tissue structure of the fish balls. This was in accordance with a previous study that the chitosan coating significantly improved the textural properties of Indian oil sardines under frozen conditions compared with the untreated samples [48].

#### 3.2.4. Whiteness

Whiteness is an index to reflect the sensory quality of frozen fish balls. As shown in Table 3, the whiteness values of both groups of samples showed a decreasing trend during seven freeze–thaw cycles. This was explained by the fact that ice crystals growing inside the fish balls caused the extrusion of muscle tissue during the freeze–thaw cycle [45], which resulted in the loss of water and nutrients and degradation in the appearance and color of the samples [49].

Before and at the 1st freeze–thaw cycles, the whiteness of the ice-coated samples decreased compared with the control group, and there was an opposite trend at the 3rd, 5th, and 7th freeze–thaw cycles. This indicated that with the increase in freeze–thaw cycles, the decreased rate of the whiteness value of the control was higher than that of the ice-coated samples, and the decrease in whiteness value of the ice-coated samples was significantly suppressed (*p* < 0.05). This could be attributed to the reduced amount of frost generation in fish balls after ice-coating, which could avoid the change in refractive index to light and thus inhibit the decrease in whiteness value during freeze–thaws. Soares et al. [50] also reported that 1.5% chitosan-coated samples performed better when maintaining salmon coloration.

#### 3.2.5. Water Status

**Figure 2:**
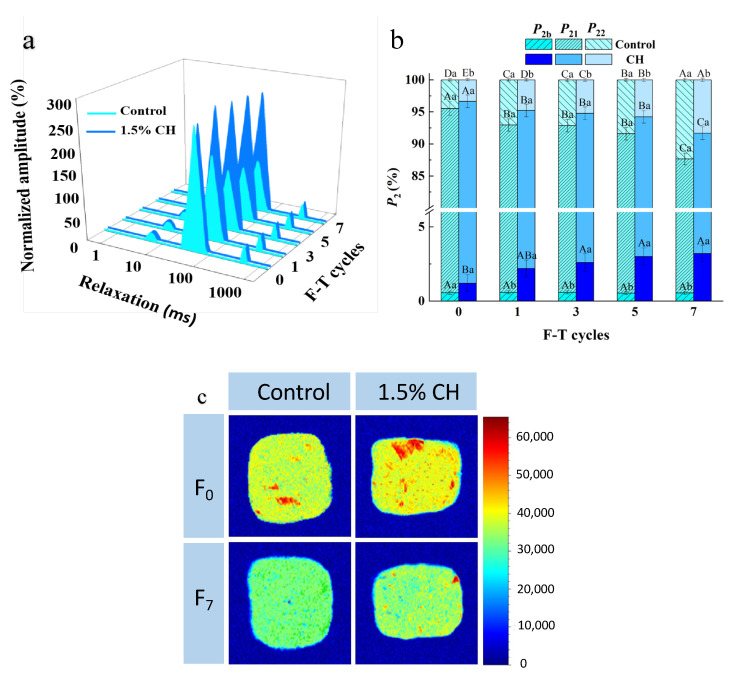
Influence of chitosan ice coating on the *T*_2_ relaxation times (**a**) and *P*_2_ (**b**) water content (**c**) of fish balls induced by freeze–thaw cycles. The means in the same treatment group with different uppercase letters (A–E) differ significantly (*p* < 0.05); the means at the same freeze–thaw cycle with different lowercase letters (a–b) differ significantly (*p* < 0.05).

Water status and content are reflected by the *T*_2_ relaxation time and P_2_ of fish balls by LF-NMR [51]. As shown in Figure 2a, both sets of samples showed three water distribution clusters: *T*_2b_ (0–10 ms) indicates bound water, which firmly adheres to the macromolecules; *T*_21_ (10–100 ms) indicates immobile water, which is present inside the high-density histone; and *T*_22_ (100–1000 ms) indicates free water, which is present outside the reticulum of myogenic fibers [52].

As shown in Table 4, the *T*_21_ and *T*_22_ values of the two sample groups increased with the number of freeze–thaws, while the *T*_2b_ values did not change significantly, which was due to the tight binding of bound water to the proteins inside the samples with a low mobility. The increase in *T*_21_ and *T*_22_ indicated that the water molecules were loosely bound. The formation of extracellular ice crystals in fish balls during thawing led to the extrusion of muscle tissue, which caused the migration and loss of internal water in the samples [53]. Cao et al. [54] reported a similar study in which freeze–thaw cycles prolonged the relaxation time, which indicated the transition from fixed water to free water in bovine rumen smooth muscle, leading to an increase in free water mobility.

In the same freeze–thaw cycle, the increase in both *T*_21_ and *T*_22_ was significantly inhibited in the 1.5% CH-coated samples compared with the control group (*p* < 0.05). At the 7th freeze–thaw cycle, the *T*_21_ and *T*_22_ of the samples after 1.5% CH coating decreased by 7.2% and 17.4%, respectively. This phenomenon was due to the fact that the CH coating technique separated the quick-frozen fish balls from the external environment through the ice coating layer, which inhibited the water migration from the interior of the samples to the surface by utilizing its excellent water retention.

As shown in Figure 2b, there was no obvious change in *P*_2b_ for both groups of samples during the whole freeze–thaw cycle, while *P*_21_ decreased significantly and *P*_22_ increased significantly (*p* < 0.05). This was caused by the increase in the number of freeze–thaws that converted the water inside the fish balls from immobile water to free water, which reduced the WHC of the product. This is consistent with the results on the amount of frost production and WHC of fish balls (Table 2). Compared with the control group, the decrease in *P*_21_ and the increase in *P*_22_ were both suppressed, which was due to the fact that the CH coating restricted the migration of water inside the samples, so that the bound water and the immobile water inside the fish balls were retained to a greater extent. 

#### 3.2.6. Water Distribution

The color and its distribution in Figure 2c can reflect the content and distribution situation of the water of the samples. The brighter the image and the more red components, the stronger the proton density of the sample and the higher the moisture content [24]. According to the figure, after seven freeze–thaw cycles, the red areas of the two groups of samples gradually changed to blue, demonstrating that water migration and loss occurred in quick-frozen fish balls. In the same freeze–thaw cycle, samples after 1.5% CH showed a stronger hydrogen proton density and higher moisture content compared with the control group. This result indicated that the ice coating treatment could inhibit the internal water migration and loss of quick-frozen fish balls during freeze–thaw cycles in order to enhance their WHC.

#### 3.2.7. Microstructure

**Figure 3:**
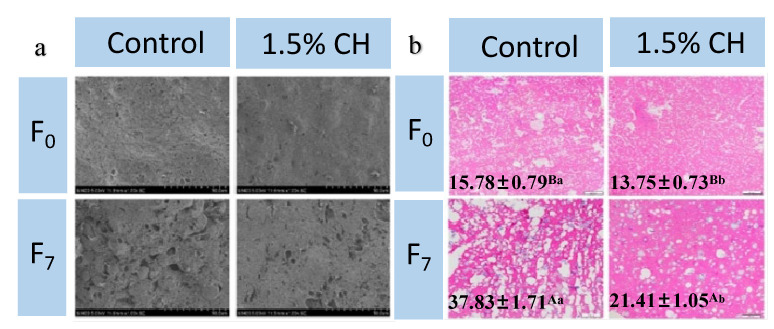
Influence of chitosan ice coating on the scanning electron microscopy (SEM) images (**a**) and optical microscope images (**b**) of fish balls induced by freeze–thaw cycles (magnification: 1000×). F_0_: 0 freeze–thaw cycle; F_7_: 7 freeze–thaw cycles. Means with different capital letters in the same treatment group (A–B) differed significantly (*p* < 0.05); means with different lowercase letters in the same freeze–thaw cycle (a–b) differed signif-icantly (*p* < 0.05).

The microstructure of the quick-frozen fish balls cut at the surface during freeze–thaw cycles is shown in Figure 3a. Before the first freeze–thaw cycle, the gel mesh structure of both sets of fresh samples were intact, and the water inside the samples had not been frozen into ice crystals. The microscopic cut surface structure was flat with small and dense pores [55]. After 7 freeze–thaw cycles, the microscopic mesh structure was severely damaged, and the denseness and flatness of the tissue were degraded. Jeong et al. [56] also showed that large and inhomogeneous ice crystals that formed inside the samples during freeze–thaw cycles severely damaged the muscle cells and caused changes in the microstructure of the samples.

On the contrary, the cut surfaces of the samples after 1.5% CH coating were slightly uneven, with small and dense pores compared with the control group, indicating that the CH coating could effectively protect the gel network structure of the surimi, thus inhibiting the physical damage caused by the generation of ice crystals during freeze–thaw cycles.

#### 3.2.8. Ice Crystal Morphology 

The size and number of ice crystals inside frozen fish products play a decisive role in the quality of the product [57]. Figure 3b shows the results of the changes in the morphology and diameters of the ice crystals inside the quick-frozen fish balls under repeated freeze–thaw conditions, which are reflected by the area of the muscle tissue (purple part) and the traces left after the generation of ice crystals (white part) of fish balls.

It can be seen that the ice crystals produced in the two groups of fresh samples were uniform in size and that the water was uniformly distributed in the samples before the first freeze–thaw cycle. After seven freeze–thaw cycles, the number and size of ice crystals increased in the two groups of samples, and the tissue surface was damaged. The ice crystal diameter of the two groups of samples showed an increase of 139.73% and 55.71% after 7 freeze–thaw cycles. This was because temperature fluctuations caused the ice crystals to crystallize, and the melted ice crystals would recollect on the large ice crystals to generate larger ice crystal apertures, thus causing the extrusion loss of water inside the samples to precipitate out to the surface [1].

Compared with the control group, the CH-coated sample showed less damage caused by ice crystal extrusion and the muscle tissue structure was dense and intact. A significant decrease of 12.86% and 43.40% in the diameter of ice crystals occurred in the coated samples before the first and at the 7th cycles. This phenomenon indicated that 1.5% CH could effectively inhibit the ice crystals’ formation and growth, and improve the quality of quick-frozen fish balls. Gutiérrez et al. [58] studied the growth of ice crystals inside the tilapia under different temperature fluctuations and found that the greater the temperature fluctuations, the larger the diameter of ice crystals generated inside the sample and the more severe the juice loss caused.

#### 3.2.9. Total Volatile Base Nitrogen (TVB-N)

**Figure 4:**
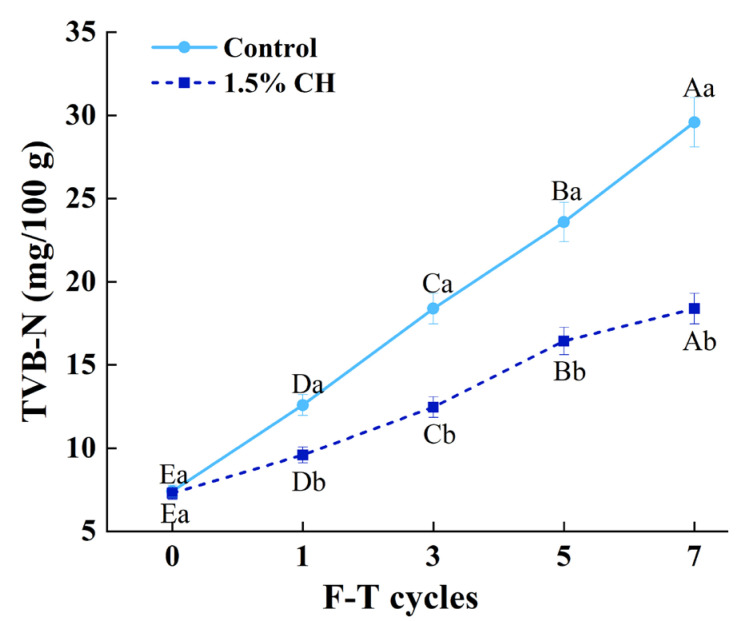
Influence of chitosan ice coating on the TVB-N of fish balls induced by freeze–thaw cycles. Means with different capital letters in the same treatment group (A–E) differed significantly (*p* < 0.05); means with different lowercase letters in the same freeze–thaw cycle (a–b) differed significantly (*p* < 0.05).

Fish balls are destroyed and amines are formed during the storage of quick-freeze fish balls as a result of the combined activity of microorganisms and endogenous enzymes. TVB-N is an important index for detecting the deterioration of fish products [59]. The effect of CH coating on the TVB-N of quick-frozen fish balls during freeze–thaw cycles is shown in Figure 4.

The TVB-N of two groups of samples increased significantly (*p* < 0.05) with the number of freeze–thaws. The increase in TVB-N values was due to the generation of autolysis in surimi and contamination of fish balls by microorganisms in the environment, leading to the degradation of proteins and some non-protein nitrogenous compounds in fish balls [60]. At the 7th freeze–thaw cycle, the TVB-N value of the sample without ice coating increased from 7.81 mg/100g to 30.30 mg/100 g, beyond the maximum acceptable value (30 mg/100 g), which indicated the spoilage of quick-frozen fish balls.

At the same freeze–thaw cycle, the increase in TVB-N values was significantly (*p* < 0.05) inhibited in the CH-coated samples compared with the control group. The TVB-N values of the CH-coated sample decreased by 23.80%, 32.21%, 30.33%, and 52.10% during cycles 1, 3, 5, and 7, respectively. This was because ice-coating technology separated the sample from the external environment, thus inhibiting the contamination of the sample by microorganisms. Meanwhile, CH as a polysaccharide has certain WHC and antibacterial effects, which can reduce the occurrence of autolysis of surimi and inhibit the increase in the TVB-N value of quick-frozen fish balls. Huang et al. [61] also reported that the TVB-N of 1.5% CH white shrimp underwent a slight increase compared with the control group.

## 4. Conclusions

In summary, with increasing the freeze–thaw cycles, ice crystal formation, melting, and recrystallization inside the quick-frozen fish balls caused physical damage to the muscle tissue, resulting in a decrease in WHC and textural properties. A comparative study of CH-coated and uncoated samples revealed that the 1.5% CH coating effectively inhibited the generation of frost on the surface of the quick-frozen fish balls compared with the control group, resulting in better color and texture of the fish balls and effectively alleviating the reduction in freshness value. In addition, the occurrence of ice crystal growth and recrystallization in the freeze–thaw cycle was inhibited, and the damage to the muscle tissue of the fish balls and the loss of free water were reduced. Hence, 1.5% CH ice coating could effectively inhibit the quality deterioration of quick-frozen fish balls during the freeze–thaw cycle. This study will serve as a guide for regulating and extending the shelf life of frozen surimi seafood.

## Figures and Tables

**Table 1:** Characterization of the physical and chemical properties of the ice solution and coating of fish balls.

CH Concentration (%)	Viscosity (cP)	Ice Coating Rate (%)	WVP (kg/m·d)	Water Solubility (%)	Transmittance (500 nm) (%)
0.5	33 ± 1 ^B^	2.90 ± 1.2 ^C^	0.031 ± 0.001 ^A^	38.96 ± 1.77 ^A^	95.5 ± 3.58 ^A^
1.0	80 ± 3 ^B^	5.2 ± 1.8 ^C^	0.018 ± 0.001 ^B^	35.85 ± 1.64 ^B^	90.8 ± 3.44 ^AB^
1.5	132 ± 5 ^B^	9.2 ± 1.2 ^B^	0.010 ± 0.001 ^C^	20.00 ± 0.97 ^C^	85.6 ± 3.21 ^BC^
2.0	6864 ± 341 ^A^	31.9 ± 1.2 ^A^	0.019 ± 0.001 ^B^	19.69 ± 0.93 ^C^	79.6 ± 2.97 ^C^

CH: chitosan. The means with different uppercase letters (A–C) in the same solution differ significantly (*p* < 0.05).

**Table 2:** Influence of chitosan ice coating on frost production and WHC of fish balls during freeze–thaw cycles.

Freeze–Thaw Cycles	Treatments	Frost Production (%)	WHC (%)		
			Thawing Loss	Cooking Loss	Centrifugal Loss
0	Control	-	-	5.21 ± 0.26 ^Ea^	5.44 ± 0.31 ^Da^
	1.5% CH	-	-	5.26 ± 0.25 ^Ea^	5.36 ± 0.29 ^Da^
1	Control	0.34 ± 0.01 ^Da^	9.01 ± 0.42 ^Da^	7.91 ± 0.39 ^Da^	9.74 ± 0.51 ^Ca^
	1.5% CH	0.03 ± 0.01 ^Db^	7.56 ± 0.41 ^Db^	6.13 ± 0.21 ^Db^	8.02 ± 0.35 ^Cb^
3	Control	2.31 ±0.11 ^Ca^	13.11 ± 0.63 ^Ca^	10.12 ± 0.50 ^Ca^	15.13 ± 0.72 ^Ba^
	1.5% CH	1.05 ± 0.05 ^Cb^	11.01 ± 0.48 ^Cb^	8.15 ± 0.39 ^Cb^	11.43 ± 0.44 ^Bb^
5	Control	5.73 ± 0.25 ^Ba^	18.89 ± 0.80 ^Ba^	13.63 ± 0.60 ^Ba^	17.48 ± 0.86 ^Aa^
	1.5% CH	2.95 ± 0.13 ^Bb^	14.21 ± 0.71 ^Bb^	9.34 ± 0.35 ^Bb^	13.74 ± 0.72 ^Ab^
7	Control	13.57 ± 0.62 ^Aa^	27.12 ± 1.40 ^Aa^	20.15 ± 1.00 ^Aa^	18.21 ± 1.53 ^Aa^
	1.5% CH	8.12 ± 0.39 ^Ab^	19.12 ± 0.81 ^Ab^	15.23 ± 0.70 ^Ab^	14.03 ± 1.02 ^Ab^

The mean values of different capital letters (A–E) in the same treatment differed significantly (*p* < 0.05); the mean values of different lowercase letters (a–b) in the same freeze–thaw cycle differed significantly (*p* < 0.05).

**Table 3:** Influence of chitosan ice coating on the texture properties and whiteness of fish balls induced by freeze–thaw cycles.

Freeze–Thaw Cycles	Treatments	Springiness (mm)	Cohesiveness	Chewiness (g)	Whiteness
0	Control	0.94 ± 0.04 ^Aa^	0.60 ± 0.03 ^Aa^	289 ± 14 ^Ab^	69.7 ± 3.4 ^Aa^
	1.5% CH	0.95 ± 0.04 ^Aa^	0.63 ± 0.03 ^Aa^	295 ± 15 ^Aa^	66.3 ± 3.2 ^Ab^
1	Control	0.63 ± 0.03 ^Bb^	0.30 ± 0.02 ^Bb^	257 ± 13 ^Bb^	68.5 ± 3.3 ^Aa^
	1.5% CH	0.89 ± 0.03 ^Ba^	0.57 ± 0.02 ^Ba^	286 ± 13 ^ABa^	65.2 ± 3.1 ^Ab^
3	Control	0.47 ± 0.02 ^Cb^	0.26 ± 0.01 ^Cb^	242 ± 12 ^BCb^	60.5 ± 2.9 ^Bb^
	1.5% CH	0.84 ± 0.03 ^BCa^	0.54 ± 0.02 ^Ba^	283 ± 14 ^ABa^	61.1 ± 3.0 ^Aa^
5	Control	0.41 ± 0.02 ^Db^	0.24 ± 0.01 ^CDb^	221 ± 11 ^Cb^	52.8 ± 2.5 ^Cb^
	1.5% CH	0.80 ± 0.03 ^Ca^	0.49 ± 0.02 ^Ca^	261 ± 13 ^BCa^	55.7 ± 2.5 ^Ba^
7	Control	0.39 ± 0.02 ^Db^	0.22 ± 0.01 ^Db^	198 ± 10 ^Db^	47.4 ± 2.2 ^Db^
	1.5% CH	0.72 ± 0.03 ^Da^	0.39 ± 0.02 ^Da^	250 ± 12 ^Ca^	50.3 ± 2.4 ^Ca^

The means with different uppercase letters in the same treatment group (A–D) differed significantly (*p* < 0.05); the means with different lowercase letters in the same freeze–thaw cycle (a–b) differed significantly (*p* < 0.05).

**Table 4:** Influence of chitosan ice coating on the *T*_2_ relaxation times of fish balls induced by freeze–thaw cycles.

Freeze–Thaw Cycles	Treatments	*T*_2_ Relaxation Time (ms)		
		*T*_2b_	*T*_21_	*T*_22_
0	Control	5.97 ± 0.27 ^Ba^	60.2 ± 3.0 ^Da^	512.8 ± 25.5 ^Ba^
	1.5% CH	5.21 ± 0.25 ^Cb^	50.2 ± 2.5 ^Db^	507.2 ± 25.1 ^Ab^
1	Control	6.20 ± 0.29 ^Ba^	63.5 ± 3.1 ^CDa^	518.1 ± 26.0 ^Ba^
	1.5% CH	5.42 ± 0.26 ^Cb^	56.4 ± 2.7 ^Cb^	509.5 ± 25.1 ^Ab^
3	Control	6.78 ± 0.32 ^Aa^	68.1 ± 3.4 ^BCa^	520.0 ± 25.8 ^Ba^
	1.5% CH	6.77 ± 0.33 ^Aa^	62.5 ± 3.0 ^Bb^	512.1 ± 25.2 ^Ab^
5	Control	6.94 ± 0.35 ^Aa^	71.7 ± 3.4 ^ABa^	521.3 ± 26.0 ^Ba^
	1.5% CH	6.83 ±0.34 ^Aa^	66.0 ± 3.2 ^ABb^	512.5 ± 25.5 ^Ab^
7	Control	7.05 ± 0.34 ^Aa^	75.1 ± 3.7 ^Aa^	622.7 ± 30.7 ^Aa^
	1.5% CH	6.01 ± 0.29 ^Bb^	69.7 ± 3.4 ^Ab^	514.0 ± 25.5 ^Ab^

The difference in means (A–D) with different uppercase letters in the same treatment group was significant (*p* < 0.05); the difference in means (a–b) with different lowercase letters in the same freeze–thaw cycle was significant (*p* < 0.05).

## Data Availability

The data presented in this study are available in the article.

## References

[1] Zheng M., Hong J., Chuai P., Chen Y., Ni H., Li Q., Jiang Z. (2022). Impacts of agar gum and fucoidan on gel properties of surimi products without phosphate. Food Sci. Nutr..

[2] Du X., Wang B., Li H., Liu H., Shi S., Feng J., Pan N., Xia X. (2022). Research progress on quality deterioration mechanism and control technology of frozen muscle foods. Compr. Rev. Food Sci. Food Saf..

[3] Sampels S. (2015). The effects of processing technologies and preparation on the final quality of fish products. Trends Food Sci. Technol..

[4] Zhang M., Niu H., Chen Q., Xia X., Kong B. (2018). Influence of ultrasound-assisted immersion freezing on the freezing rate and quality of porcine longissimus muscles. Meat Sci..

[5] Nuerjiang M., Li Y., Yue X., Kong B., Liu H., Wu K., Xia X. (2023). Analysis of inhibition of guava (*Psidium guajava l.*) leaf polyphenol on the protein oxidative aggregation of frozen chicken meatballs based on structural changes. Food Res. Int..

[6] Olatunde O.O., Benjakul S. (2018). Natural preservatives for extending the shelf-life of seafood: A revisit. Compr. Rev. Food Sci. Food Saf..

[7] Li H., Bai X., Li Y., Du X., Wang B., Li F., Shi S., Pan N., Zhang Q., Xia X. (2022). The positive contribution of ultrasound technology in muscle food key processing and its mechanism-a review. Crit. Rev. Food Sci. Nutr..

[8] Kang T., Lee D., Ko Y., Jun S. (2022). Effects of pulsed electric field (*PEF*) and oscillating magnetic field (*OMF*) on supercooling preservation of beef at different fat levels. Int. J. Refrig..

[9] Otero L., Martino M., Zaritzky N., Solas M., Sanz P.D. (2000). Preservation of microstructure in peach and mango during high-pressure-shift freezing. J. Food Sci..

[10] Islam M.N., Zhang M., Adhikari B., Xinfeng C., Xu B.G. (2014). The effect of ultrasound-assisted immersion freezing on selected physicochemical properties of mushrooms. Int. J. Refrig..

[11] Zhu S., Yu J., Chen X., Zhang Q., Cai X., Ding Y., Zhou X., Wang S. (2021). Dual cryoprotective strategies for ice-binding and stabilizing of frozen seafood: A review. Trends Food Sci. Technol..

[12] Tavares L., Souza H.K., Gonçalves M.P., Rocha C.M. (2021). Physicochemical and microstructural properties of composite edible film obtained by complex coacervation between chitosan and whey protein isolate. Food Hydrocoll..

[13] Hosseini S.F., Rezaei M., Zandi M., Ghavi F.F. (2013). Preparation and functional properties of fish gelatin–chitosan blend edible films. Food Chem..

[14] Fan L., Ruan D., Shen J., Hu Z., Liu C., Chen X., Xia W., Xu Y. (2022). The role of water and oil migration in juiciness loss of stuffed fish ball with the fillings of pig fat/meat as affected by freeze-thaw cycles and cooking process. LWT Food Sci. Technol..

[15] Cho W.Y., Yeon S.J., Hong G.E., Kim J.H., Tsend-Ayush C., Lee C.H. (2017). Antioxidant activity and quality characteristics of yogurt added green olive powder during storage. Korean J. Food Sci. Anim. Resour..

[16] Sundararajan S., Prudente A., Bankston J.D., King J.M., Wilson P., Sathivel S. (2011). Evaluation of green tea extract as a glazing material for shrimp frozen by cryogenic freezing. J. Food Sci..

[17] Wildan M.W., Lubis F.I. (2021). Fabrication and characterization of chitosan/cellulose nanocrystal/glycerol bio-composite films. Polymers.

[18] Urquiola A., Alvarez G., Flick D. (2017). Frost formation modeling during the storage of frozen vegetables exposed to temperature fluctuations. J. Food Eng..

[19] Bai X., Shi S., Kong B., Chen Q., Liu Q., Li Z., Wu K., Xia X. (2023). Analysis of the influencing mechanism of the freeze–thawing cycles on in vitro chicken meat digestion based on protein structural changes. Food Chem..

[20] Shi T., Wang X., Li M., Xiong Z., McClements D.J., Bao Y., Song T., Li J., Yuan L., Jin W. (2022). Mechanism of low-salt surimi gelation induced by microwave heating combined with L-arginine and transglutaminase: On the basis of molecular docking between L-arginine and myosin heavy chain. Food Chem..

[21] Lv Y., Xie J. (2022). Quality of Cuttlefish as Affected by Different Thawing Methods. Int. J. Food Prop..

[22] Gao Y., Li M., Zhang L., Wang Z., Yu Q., Han L. (2021). Preparation of rapeseed oil oleogels based on beeswax and its application in beef heart patties to replace animal fat. LWT Food Sci. Technol..

[23] Jia N., Kong B., Liu Q., Diao X., Xia X. (2012). Antioxidant activity of black currant (*Ribes nigrum L.*) extract and its inhibitory effect on lipid and protein oxidation of pork patties during chilled storage. Meat Sci..

[24] Nian L., Cao A., Cai L., Ji H., Liu S. (2019). Effect of vacuum impregnation of red sea bream (*Pagrosomus major*) with herring AFP combined with CS@ Fe_3_O_4_ nanoparticles during freeze-thaw cycles. Food Chem..

[25] Lan W., Hu X., Sun X., Zhang X., Xie J. (2020). Effect of the number of freeze-thaw cycles number on the quality of Pacific white shrimp (*Litopenaeus vannamei*): An emphasis on moisture migration and microstructure by LF-NMR and SEM. Aquacult. Fish..

[26] Sun Q., Sun F., Xia X., Xu H., Kong B. (2019). The comparison of ultrasound-assisted immersion freezing, air freezing and immersion freezing on the muscle quality and physicochemical properties of common carp (*Cyprinus carpio*) during freezing storage. Ultrason. Sonochem..

[27] Sun Q., Chen Q., Li F., Zheng D., Kong B. (2016). Biogenic amine inhibition and quality protection of Harbin dry sausages by inoculation with Staphylococcus xylosus and Lactobacillus plantarum. Food Control.

[28] Do Amaral Sobral P.J., Gebremariam G., Drudi F., De Aguiar Saldanha Pinheiro A.C., Romani S., Rocculi P., Dalla Rosa M. (2022). Rheological and Viscoelastic Properties of Chitosan Solutions Prepared with Different Chitosan or Acetic Acid Concentrations. Foods.

[29] Farajzadeh F., Motamedzadegan A., Shahidi S.A., Hamzeh S. (2016). The effect of chitosan-gelatin coating on the quality of shrimp (*Litopenaeus vannamei*) under refrigerated condition. Food Control.

[30] TR EAEU 040/2016: Technical Regulation of the Eurasian Customs Union “On the Safety of Fish and Fish Products”. https://eacgroupcompany.com/en/regulations/trcu040-2016.

[31] TR EAEU 021/2011: Technical Regulation of the Eurasian Customs Union “About Food Safety”. https://eacgroupcompany.com/en/regulations/trcu021-2011.

[32] Duborasova T., Kolobov S., Osipenko E. (2020). Improving the properties of frozen shrimp by improving the characteristics of the surface ice glaze. E3S Web Conf..

[33] Vanhaecke L., Verbeke W., De Brabander H.F. (2010). Glazing of frozen fish: Analytical and economic challenges. Anal. Chim. Acta.

[34] Ren L., Yan X., Zhou J., Tong J., Su X. (2017). Influence of chitosan concentration on mechanical and barrier properties of corn starch/chitosan films. Int. J. Biol. Macromol..

[35] Roy S., Rhim J.W. (2021). Fabrication of chitosan-based functional nanocomposite films: Effect of quercetin-loaded chitosan nanoparticles. Food Hydrocoll..

[36] Meléndez-Pérez R., Rodríguez-Hernández Y., Arjona-Román J.L., Méndez-Albores A., Coria-Hernández J. (2022). Frost Formation in Frozen Meat Packaged with Two Plastic Films (LDPE and PVC). Processes.

[37] Wu C., Fu S., Xiang Y., Yuan C., Hu Y., Chen S., Liu D., Ye X. (2016). Effect of Chitosan Gallate coating on the quality maintenance of refrigerated (4 °C) silver pomfret (*Pampus argentus*). Food Bioprocess Technol..

[38] Li D., Jia S., Zhang L., Wang Z., Pan J., Zhu B., Luo Y. (2017). Effect of using a high voltage electrostatic field on microbial communities, degradation of adenosine triphosphate, and water loss when thawing lightly-salted, frozen common carp (*Cyprinus carpio*). J. Food Eng..

[39] Wang B., Li F., Pan N., Kong B., Xia X. (2021). Effect of ice structuring protein on the quality of quick-frozen patties subjected to multiple freeze-thaw cycles. Meat Sci..

[40] Pan N., Dong C., Du X., Kong B., Sun J., Xia X. (2021). Effect of freeze-thaw cycles on the quality of quick-frozen pork patty with different fat content by consumer assessment and instrument-based detection. Meat Sci..

[41] Flórez M., Guerra-Rodríguez E., Cazón P., Vázquez M. (2022). Chitosan for food packaging: Recent advances in active and intelligent films. Food Hydrocoll..

[42] Kauffman R.G., Greaser M.L., Pospiech E., Russell R.L. (1997). Method of Improving the Water-Holding Capacity, Color, and Organoleptic Properties of Beef, Pork, and Poultry. U.S. Patent.

[43] Lv Y., Chu Y., Zhou P., Mei J., Xie J. (2021). Effects of different freezing methods on water distribution, microstructure and protein properties of cuttlefish during the frozen storage. Appl. Sci..

[44] Nawaz T., Fatima M., Shah S.Z.H., Afzal M. (2020). Coating effect of rosemary extract combined with chitosan on storage quality of mori (*Cirrhinus mrigala*). J. Food Process. Preserv..

[45] Souissi N., Jridi M., Nasri R., Slama R.B., Njeh M., Nasri M. (2016). Effects of the edible cuttlefish gelatin on textural, sensorial and physicochemical quality of octopus sausage. LWT Food Sci. Technol..

[46] Wang Y., Miyazaki R., Saitou S., Hirasaka K., Takeshita S., Tachibana K., Taniyama S. (2018). The effect of ice crystals formations on the flesh quality of frozen horse mackerel (*Trachurus japonicus*). J. Texture Stud..

[47] Lv Y., Xie J. (2021). Effects of freeze–thaw cycles on water migration, microstructure and protein oxidation in cuttlefish. Foods.

[48] Mohan C.O., Ravishankar C.N., Lalitha K.V., Gopal T.S. (2012). Effect of chitosan edible coating on the quality of double filleted Indian oil sardine (*Sardinella longiceps*) during chilled storage. Food Hydrocoll..

[49] Filgueras R.S., Gatellier P., Zambiazi R.C., Santé-Lhoutellier V. (2011). Effect of frozen storage duration and cooking on physical and oxidative changes in M. Gastrocnemius pars interna and M. Iliofiburalis of rhea americana. Meat Sci..

[50] Soares N.M., Oliveira M.S., Vicente A.A. (2015). Effects of glazing and chitosan-based coating application on frozen salmon preservation during six-month storage in industrial freezing chambers. LWT Food Sci. Technol..

[51] Tan M., Lin Z., Zu Y., Zhu B., Cheng S. (2018). Effect of multiple freeze-thaw cycles on the quality of instant sea cucumber: Emphatically on water status of by LF-NMR and MRI. Food Res. Int..

[52] Li F., Zhong Q., Kong B., Wang B., Pan N., Xia X. (2020). Deterioration in quality of quick-frozen pork patties induced by changes in protein structure and lipid and protein oxidation during frozen storage. Food Res. Int..

[53] Nikoo M., Benjakul S. (2015). Potential application of seafood-derived peptides as bifunctional ingredients, antioxidant–cryoprotectant: A review. J. Funct. Foods.

[54] Cao Y., He S., Yu Q., Han L., Zhang W., Zou X. (2022). Effects of multiple freeze–thaw cycles on meat quality, nutrients, water distribution and microstructure in bovine rumen smooth muscle. Int. J. Food Sci. Technol..

[55] Chen X., Li X., Yang F., Wu J., Huang D., Huang J., Wang S. (2022). Effects and mechanism of antifreeze peptides from silver carp scales on the freeze-thaw stability of frozen surimi. Food Chem..

[56] Jeong J.Y., Kim G.D., Yang H.S., Joo S.T. (2011). Effect of freeze–thaw cycles on physicochemical properties and color stability of beef semimembranosus muscle. Food Res. Int..

[57] Chevalier D., Sequeira-Munoz A., Le Bail A., Simpson B.K., Ghoul M. (2000). Effect of freezing conditions and storage on ice crystal and drip volume in turbot (*Scophthalmus maximus*): Evaluation of pressure shift freezing vs. air-blast freezing. Innov. Food Sci. Emerg. Technol..

[58] Gutiérrez M.S.C., Oliveira C.M.D., Melo F.R., Silveira V. (2017). Limit growth of ice crystals under different temperature oscillations levels in nile Tilapia. Food Sci. Technol..

[59] Shi S., Xu X., Feng J., Ren Y., Bai X., Xia X. (2023). Preparation of NH_3_- and H_2_S- sensitive intelligent pH indicator film from sodium alginate/black soybean seed coat anthocyanins and its use in monitoring meat freshness. Food Packag. Shelf Life.

[60] Mousakhani-Ganjeh A., Hamdami N., Soltanizadeh N. (2015). Impact of high voltage electric field thawing on the quality of frozen tuna fish (*Thunnus albacares*). J. Food Eng..

[61] Huang J., Chen Q., Qiu M., Li S. (2012). Chitosan-based edible coatings for quality preservation of postharvest whiteleg shrimp (*Litopenaeus vannamei*). J. Food Sci..

